# Clinical Features and Follow-Up of Prurigo Pigmentosa: A Case Series

**DOI:** 10.7759/cureus.24600

**Published:** 2022-04-29

**Authors:** Muazzez Cigdem Oba, Cigdem Dicle Arican

**Affiliations:** 1 Department of Dermatology, Sancaktepe Sehit Prof. Dr. Ilhan Varank Research and Traning Hospital, Istanbul, TUR; 2 Department of Pathology, Sancaktepe Sehit Prof. Dr. Ilhan Varank Research and Training Hospital, Istanbul, TUR

**Keywords:** prurigo pigmentosa of nagashima, prurigo pigmentosa, oral doxycycline, ketosis, diet

## Abstract

Introduction

Prurigo pigmentosa (PP) is an underrecognized disease in the Western population. Our aim is to describe the clinical features and follow-up outcomes of Caucasian patients diagnosed with PP.

Methods

This case series was conducted in the dermatology outpatient clinic of a tertiary hospital. Patients with confirmed PP from May 2020 to June 2021 were included in the study. Patient demographics, clinical features, potential triggers, treatment and follow-up data were recorded.

Results

A total of eight patients with female predominance were identified. The mean age of the patients was 24.5. The duration of symptoms ranged from four days to six months. All patients presented with pruritic, papular or papulovesicular lesions. Net-like hyperpigmentation was also present at the initial visit in two patients, in whom the duration of the symptoms was the longest. Lesions were most commonly located on the chest and back. Six of eight patients reported alteration of diet that potentially led to ketosis. Doxycycline 200 mg daily for two weeks led to a complete response in all six medically treated patients. Duration of follow-up ranged from 1-14 months (mean: 7.2 months). In five patients with a follow-up duration of more than three months, postinflammatory hyperpigmentation was resolved without any treatment. Only one patient had a recurrence.

Conclusion

PP does not seem to be a rare disease. Young women are most commonly affected, and ketosis stemming from decreased calorie intake may be the etiological factor in the majority of the patients. Dermatologists should be familiar with early signs of PP in order to minimize unnecessary therapies, recurrences and long-lasting hyperpigmentation.

## Introduction

Prurigo pigmentosa (PP), AKA Nagashima’s disease, is a rare inflammatory dermatosis of unknown etiology. The disease was first reported in Japan in 1971, with a growing number of publications in recent decades [1,2]. However, to date, most of the reports describe PP in patients of East Asian descent [2]. Therefore, in this study, we aimed to define clinical features and follow-up outcomes of PP in a series of Caucasian patients.

## Materials and methods

This case series was conducted in the dermatology outpatient clinic of Sancaktepe Sehit Prof. Dr. Ilhan Varank Research and Training Hospital, Istanbul, Turkey. Patients with confirmed PP from May 2020 to June 2021 were included in the study. The diagnosis of PP was based on clinical findings and histopathological confirmation. Patient demographics (age and sex) and seasonal distribution of cases, clinical features (duration of symptoms, history of recurrent rash, family history of PP, elementary lesions, presence of pruritus, location of lesions), comorbidities and potential triggers, treatment and follow-up data (duration of follow-up, recurrences if any) were recorded.

## Results

A total of eight patients were diagnosed clinically and histologically as PP in the study period (Table 1).

**Table 1:** Demographics and clinical features of the patients CR: complete resolution PIH: postinflammatory hyperpigmentation

Patient	Age/Sex/race	Clinical findings	Duration	Potential trigger	Treatment	Follow-up
1	15/M/white	Itchy urticarial papules in reticulated pattern on the chest	7 days	Fasting for 15 hours per day	Doxycycline 200mg/day with CR in 14 days	No recurrence at 14 months, no PIH
2	22/F/white	Itchy urticarial papules in reticulated pattern along mid-back and intermammary area	7 days	Ketogenic diet	Doxycycline 200mg/day with CR in 14 days	No recurrence at 14 months, no PIH
3	40/F/white	Itchy scattered, crusted erythematous papules and net-like hyperpigmentation in intermammary area	6 months	Lactating	None, spontaneous resolution	PIH, no recurrence at 8 months
4	24/F/white	Itchy papules at sides and nape of the neck, supraclavivular and intermammary area	4 days	Lowering of carbohydrates in diet	Doxycycline 200mg/day with CR in 14 days without PIH.	One recurrence in 8 months potentially triggered by a change in diet.
5	20/F/white	Itchy papulovesicles in intermammary area	5 days	Lowering of carbohydrates in diet	Doxycycline 200mg/day with CR in 14 days, leaving postlesional erythema	No recurrence, no PIH at 7 months
6	19/F/white	Itchy crusted erythematous papules, net-like hyperpigmentation on intermammary area and abdomen	One month	None	Doxycycline 200mg/day with CR in 14 days	No recurrence at 4 month, PIH
7	30/F/white	Itchy urticarial papules on the mid-upper back	10 days	Lowering of carbohydrates in diet	None, spontaneous resolution with cessation of diet	No recurrence at 2 months, PIH
8	26/M/white	Itchy reticulated and confluent erythematous papules on the chest and back	14 days, recurrent	Lowering of carbohydrates in diet, protein supplementation	Doxycycline 200mg/day with CR in 14 days leaving PIH	No recurrence at 1 month, PIH

Patient demographics and seasonal distribution of cases

The mean age of the patients was 24.5 years (range: 15-40 years). Female patients were predominant (six women, two men). None of the patients were pregnant; there was one lactating patient. Five of eight patients had applied to our outpatient clinic in spring while the remaining three patients had applied in winter. None of the patients had symptoms associated with COVID-19 infection such as constitutional symptoms, cough, etc., nor did they have close contact with a COVID-19-infected patient.

Clinical features

The duration of symptoms ranged from four days to six months with a mean duration of 32.1 days. Seven of the eight patients had applied during the first episode, stating they did not have a history of similar rash in the past. One patient had a history of an undiagnosed, recurrent, pruritic rash. Family history of PP was positive in patient 4, whose mother was also diagnosed with PP. All patients presented with pruritic, papular or papulovesicular lesions. All lesions were symmetrically distributed. There was accompanying net-like hyperpigmentation in two patients at presentation, in whom the duration of the symptoms was the longest (one month and six months). Lesions were most commonly located on the chest (7/8) and back (3/8). Other sites that were involved included the sides and back of the neck, supraclavicular region and upper abdomen.

Comorbidities and potential triggers

None of the patients had a comorbid systemic disease. Of note, six of eight patients reported alteration of the diet that potentially led to ketosis, before the eruption of the rash. Specifically, three patients reported restricted carbohydrate intake. One patient was on a ketogenic diet with increased fat intake. One patient was on fasting for religious reasons. And one patient had lowered carbohydrate intake and was also taking protein supplements for bodybuilding. Lactating might have contributed to the development of PP in one patient, who reported spontaneous improvement one month later when she stopped lactating. The remaining one patient had no apparent trigger.

Treatment and follow-up

**Figure 1:**
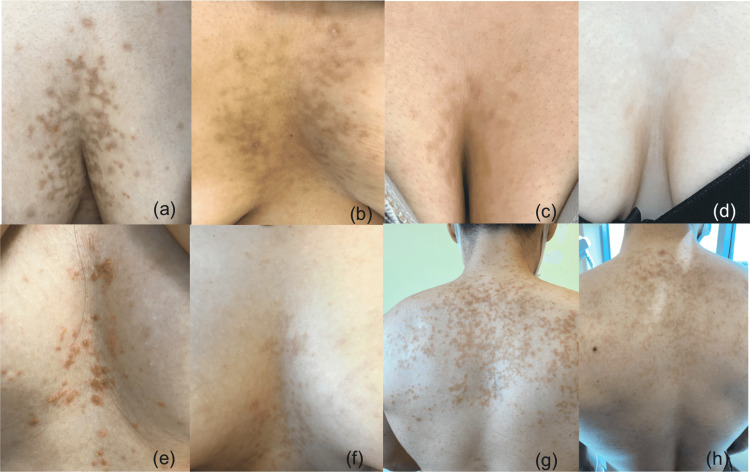
Clinical photographs of four patients (a) Patient 3 showing reticular hyperpigmentation in the intermammary area. (b) Spontaneous resolution with postinflammatory hyperpigmentation at one month. Two scars from punch biopsy can be seen. (c) Patient 4 showing erythematous, confluent papules in the intermammary area (d) Complete resolution without postinflammatory hyperpigmentation following 14 days of doxycycline therapy. (e) Patient 5 presenting with pruritic papulovesicles.  (f) Complete resolution with postlesional erythema following 14 days of doxycycline therapy. (g) Patient 8 showing confluent erythematous papules on the back. (h) Complete resolution with postinflammatory hyperpigmentation following 14 days of doxycycline therapy. Crusted hyperpigmented area is the biopsy site.

Lesions regressed without medical treatment in two patients (cessation of diet and cessation of lactating), while the remaining patients were prescribed doxycycline 200 mg daily, which led to complete response in all patients at two weeks (Figure 1). There were no side effects due to doxycycline therapy in any of the patients. Duration of follow-up ranged from 1-14 months (mean: 7.2 months). In five patients with a follow-up duration of more than three months, postinflammatory hyperpigmentation was resolved without any treatment. One patient had a recurrence associated with a change in diet, which rapidly resolved upon initiating doxycycline therapy.

## Discussion

To date, cases of PP are most frequently reported from East Asia. However, the disease was also described in other ethnicities, pointing to an underdiagnosis of the disease outside East Asia [2]. Baykal et al. had postulated that PP might not be an uncommon disease in Turkey with their series of six patients diagnosed within four years [3]. We confirmed their observation with a notably higher prevalence of eight patients in one year. In line with the literature, we observed a clear predilection of the condition for young women [2,3]. We observed a seasonal coalescence of cases in spring. This might be attributed to sweating as an exacerbating factor for PP [1].

Although the etiology of PP is not clearly understood, perivascular accumulation of ketone bodies is thought to trigger a neutrophilic infiltration [2]. There is a growing association of PP with ketosis, with 25% of patients having a history of dietary changes [2,4]. In our series, this association was striking with history of diet changes prior to development of PP in 75% of the patients. In a series of PP patients from Swiss, atopy was also found to be a predisposing factor [5]. However, in our series, none of the patients reported atopic diathesis. Other associated conditions reported include allergy to nickel, pregnancy, primary biliary cirrhosis, Sjögren’s syndrome adult onset Still’s disease and H.pylori infection [1].

As for family history of PP, it was not reported previously, except for the occurrence of PP in monozygotic twins [6]. Yet, segmentally arranged lesions of PP were reported, which may be suggestive of a genetic predisposition [7,8]. Since our series included one patient with a positive family history, we think that careful history taking and raising awareness in the patient’s family might be important.

Similar to previous reports, clinical features consisted mostly of erythematous papules in a reticulated pattern and hyperpigmentation, occurring most frequently on back and chest [2]. To our knowledge, the hyperpigmentation caused by PP is not well studied in the literature. Considering most patients with PP are young women, postinflammatory hyperpigmentation is a bothersome side effect. We observed rapid spontaneous resolution of hyperpigmentation in patients with short disease duration, whereas patients with longer histories had more long-lasting hyperpigmentation. Thus, early recognition of the disease seems to be an important factor to minimize hyperpigmentation.

Histopathologically three stages of PP have been defined. Early lesions consisting of urticarian papules show perivascular neutrophilic infiltration and spongiosis, while crusted papulovesicular lesions reveal lymphocytic infiltration and necrotic keratinocytes. In late lesions, dermal melanophages accompany lymphocytic infiltration [1]. Of note, a very recent publication suggested that PP may be classified among neutrophilic dermatoses. The authors observed immature neutrophils/myeloid precursor cells in the epidermis and dermis upon immunohistochemical examination with myeloperoxidase and CD11c stains [9].

Clinical differential diagnoses of inflammatory lesions in PP include dermatitis herpetiformis, linear IgA disease and acute lupus erythematosus, among others [1,10]. Clinically, the predilection of PP to involve the trunk and reticular distribution of the lesions are helpful in the differential diagnosis. In PP patients presenting with hyperpigmentation, the main clinical differential diagnosis is confluent and reticular papillomatosis (CARP) also known as Gougerot-Carteaud syndrome [1,2]. In contrast to PP, CARP lesions are hyperkeratotic and are not associated with pruritus [2,10]. Other diseases that should be considered include lichen planus pigmentosus, macular amyloidosis, pigmented contact dermatitis, erythema caloricum, and erythema dyschromicum perstans [1, 11].

As for management, resolution of PP has been reported with diet modification alone (as patient 7 in our series) [12,13] or treatment of hyperglycemia and dehydration [14]. Medically, oral doxycycline is considered to be the first-line treatment of PP [5], and administration of doxycycline was successful in all of the medically treated patients in our series. The plausible mechanism of action of doxycycline is the inhibition of neutrophil chemotaxis [2]. Duration of treatment is widely variable in literature varying from two weeks to 14 months [15,16]. However, in our series, a two-week course of doxycycline was sufficient to attain complete response in all patients. Other drugs that affect neutrophil migration and/or function such as minocycline and dapsone can also be used in the treatment of PP [3]. The recurrence rate after stopping therapy with all three treatments (doxycycline, minocycline and dapsone) was reported to be around 10% [2]. In our series, in a mean follow-up period of 7.2 months, only one patient had a recurrence. As another anti-inflammatory treatment targeting neutrophil chemotaxis, colchicine (1.5 g/day) was used successfully in the treatment of a PP case [17]. Systemic isotretinoin was also successful to resolve PP in two case reports [18,19]. Moreover, macrolide antibiotics, including clarithromycin and roxythromycin, can be considered alternative therapies as antibiotics with anti-inflammatory effects [20].

Limitations of our study include the retrospective design and the small sample size.

## Conclusions

Our data with eight patients diagnosed with PP over only one year in a single center, indicates that PP is not a rare disease. It is rather a probably underrecognized one for two main reasons. Firstly, the disease may resolve spontaneously, or more seemingly upon an increase in carbohydrate intake in some patients. Secondly, the condition is not readily known among dermatologists and may be misdiagnosed as insect bites, eczema, drug eruption, food allergy, confluent and reticulated papillomatosis, etc. Young women are most commonly affected, and ketosis stemming from decreased calorie intake may be the etiological factor in the majority of the patients. Lack of accompanying hyperpigmentation in early disease may lead to diagnostic challenges. Dermatologists should be familiar with early signs of PP in order to minimize unnecessary therapies, recurrences and long-lasting hyperpigmentation. Lastly, doxycycline therapy is useful in both first and recurrent attacks of the disease.
